# Draft Genome Sequence of *Bacillus velezensis* 3A-25B, a Strain with Biocontrol Activity against Fungal and Oomycete Root Plant Phytopathogens, Isolated from Grassland Soil

**DOI:** 10.1128/genomeA.01021-17

**Published:** 2017-09-28

**Authors:** Inés Martínez-Raudales, Yumiko De La Cruz-Rodríguez, Julio Vega-Arreguín, Alejandro Alvarado-Gutiérrez, Atzin Fraire-Mayorga, Miguel Alvarado-Rodríguez, Victor Balderas-Hernández, José Manuel Gómez-Soto, Saúl Fraire-Velázquez

**Affiliations:** a Biología Integrativa de Plantas y Microorganismos, Unidad Académica de Ciencias Biológicas, Universidad Autónoma de Zacatecas, Zacatecas, México; b ENES-León, Universidad Nacional Autónoma de México, León, Guanajuato, México; c Unidad Académica de Agronomía, Universidad Autónoma de Zacatecas, Zacatecas, México; d Unidad Académica de Matemáticas, Universidad Autónoma de Zacatecas, Zacatecas, México

## Abstract

Here, we present the draft genome of *Bacillus velezensis* 3A-25B, which totaled 4.01 Mb with 36 contigs, 3,948 genes, and a GC content of 46.34%. This strain, which demonstrates biocontrol activity against root rot causal phytopathogens in horticultural crops and friendly interactions in roots of pepper plantlets, was obtained from grassland soil in Zacatecas Province, Mexico.

## GENOME ANNOUNCEMENT

In conventional agriculture, the common use of pesticides is a growing concern because of the undesirable side effects in the environment, with negative impact on biodiversity, in addition to the social pressure to have innocuous food coming from agricultural production. In this context, biocontrol of phytopathogens in agrosystems is a strategy with an increasing interest. The genus *Bacillus* contains at least 336 species of bacteria (1) that are ubiquitous in nature, including water, soil, and plants, even in extreme environments (2). *Bacillus* spp. have been reported to have useful properties with agricultural purposes (3, 4). *Bacillus velezensis* is a bacterium with biocontrol activity against a number of phytopathogens and is a promoter of plant growth (5, 6).

Here, we report the draft genome of *B. velezensis* 3A-25B, isolated from soil sampled in grassland in Zacatecas, Mexico. This strain exhibits suppressing activity in confrontations against isolates of *Phytophthora capsici*, *Fusarium solani*, *Fusarium oxysporum*, and *Rhizoctonia solani*, with suppressing activity of more than 60% toward each phytopathogen. The *B. velezensis* 3A-25B strain shows an avirulent interaction in root inoculation in pepper plantlets and the induction of sesquiterpene cyclase (*CaSC1*) and PR1 (*CaBPR1*), which are both plant defense response genes (7).

The *B. velezensis* 3A-25B genome was sequenced using the Illumina MiSeq platform with a 2 × 75 paired-end run at the sequencing laboratory at the Unidad de Ciencias Biológicas, Universidad Autónoma de Zacatecas, Mexico. One nanogram of DNA was used to construct the genome libraries according to the Nextera kit instructions (Illumina, San Diego, CA, USA). The quality was examined with a Bioanalyzer 2010 (Agilent Technologies) following standard normalization, and 15 pM was used for sequencing. For the genome assembly, the SPAdes genome assembler was used (8), and the quality of the assembly was analyzed by QUAST version 4.1 software (9). The NCBI Prokaryotic Genome Annotation Pipeline was used to predict protein-coding genes, structural RNAs, and tRNAs. The assembled draft genome resulted in 4,011,370 bp, with 36 contigs and a GC content of 46.34%. It contains 3,948 total genes, including 3,786 protein-coding genes, 83 RNA genes, 10 rRNAs (8 5S, 1 16S, and 1 23S), 68 tRNAs, 5 noncoding RNAs, and 79 pseudogenes.

The predicted biological functions revealed a number of genes related to several functions, including *OppF*, a component of the operon OppABCDF involved in peptide internalization, biofilm production, adhesion, or virulence (10–12); *BacA*, which participates in the biosynthesis of the antibiotic bacilysin, a compound active against bacteria and fungal species (13); *CheA*, *CheC*, and *CheY*, implicated in chemotaxis (14); endoglucanase and *N*-acetylglucosamine-6-phosphate deacetylase, implicated in the assimilation of diverse carbon sources (15); a group of genes involved in fatty acid metabolism; and four phage-related proteins. All of the genes in this bacterium could be associated with its dynamic and successful behavior in constantly changing environments, including interacting with plants and fighting back against deleterious microorganisms. The draft genome of *B. velezensis* strain 3A-25B will contribute to increasing the knowledge of molecular mechanisms that are specific to biocontrol agents pertaining to *Bacillus* spp., reinforcing the biocontrol strategies used in agriculture in Mexico and other latitudes.

### Accession number(s).

This whole-genome shotgun project has been deposited in DDBJ/ENA/GenBank under accession number MLCW00000000. The version described in this paper is the first version, MLCW01000000.
